# Comparison and phylogenetic analysis based on the B2L gene of orf virus from goats and sheep in China during 2009-2011

**DOI:** 10.1007/s00705-013-1946-6

**Published:** 2013-12-17

**Authors:** Keshan Zhang, Yongjie Liu, Hanjin Kong, Youjun Shang, Xiangtao Liu

**Affiliations:** State Key Laboratory of Veterinary Etiological Biology, National Foot and Mouth Disease Reference Laboratory, Lanzhou Veterinary Research Institute, Chinese Academy of Agricultural Sciences, Xujiaping No. 1 Yanchangpu, Lanzhou, 730046 Gansu People’s Republic of China

## Abstract

As a zoonotic infectious disease, orf outbreaks have been reported in China in recent years. However, molecular epidemiology analysis has not been performed for Chinese orf virus (ORFV) strains. Here, we have identified 13 ORFVs from goats and sheep in China between 2009 and 2011. Thirty-four complete *B2L* sequences were used to construct a phylogenetic tree to elucidate the molecular epidemiology of ORFV in China. Nucleotide sequences of *B2L* genes of clinical samples and attenuated vaccine strains were aligned and compared. Three genotypes were found by molecular epidemiology analysis. Amino acid substitutions were dispersed among *B2* polypeptides from wild and attenuated ORFV strains.

**Electronic supplementary material:**

The online version of this article (doi:10.1007/s00705-013-1946-6) contains supplementary material, which is available to authorized users.

Ovine contagious pustular dermatitis (orf) is an infectious viral zoonosis. Diseases caused by orf virus (ORFV) occur worldwide and have been reported in many countries [13]. ORFV causes a common viral skin disease that infects a range of wild ruminant species [10] as well as humans [6, 15, 23], especially immunodeficient individuals [3]. It often infects farmers, abattoir workers, veterinarians, and sheep shearers, who are considered to be at the greatest risk due to their professions; others at high risk are those engaged in the religious slaughter of animals [10, 12, 19, 24]. Thus, orf is a serious health threat to the sheep industry as well as to humans.

ORFV belongs to the genus *Parapoxvirus* of the family *Poxviridae* [14] and has an approximately 134–139-kb linear double-stranded DNA genome [8]; the whole genome has a high GC content of approximately 63.5 % [33]. The *B2L* gene of ORFV encodes a highly immunogenic envelope protein that induces a strong antibody response [8, 26]. A polymerase chain reaction (PCR) method based on the *B2L* gene is typically used to detect ORFV [1, 13, 17, 22, 30]. Complete or partial B2L sequences have often been used in phylogenetic analysis in India [13], Korea [22], China [7, 20, 34, 36], Brazil [1], and Turkey [18].

Orf was first reported in China in 1955. From the 1980s to the 1990s, orf was detected in eight Chinese provinces including Qinghai, Gansu, Tibet, Xinjiang, Liaoning, Jiangxi, Heilongjiang, and Hebei. In recent years, orf outbreaks have occurred in 17 Chinese provinces and within the city of Beijing [34]. Furthermore, seven women and four men were infected with the ORFV in Fujian Province in 2005. Thus, orf is a nationally important zoonosis in China. Several field cases have been reported [7], but little is known about the molecular epidemiology of the ORFV isolates from China. Thus, a phylogenetic analysis of ORFV in China is urgently needed to evaluate its molecular epidemiology and distribution characteristics. A total of 14 ORFV isolates were identified in clinical samples (one from a vaccine strain) from 10 provinces between 2009 and 2011. Phylogenetic analysis of the ORFV strains was performed based on the complete *B2L* gene sequence data from China and other countries deposited in GenBank (Table 1). We aligned and compared the deduced *B2L* amino acid sequences from the ORFV strains isolated from the clinical samples and attenuated vaccine strains. This is the first systematic phylogenetic analysis of orf virus in China, and the results may help to elucidate the molecular characteristics of ORFV in China or even worldwide.

**Table 1:** Detailed information about the *B2L* sequences of the orf virus (ORFV) strains used in the study

No.	Virus strain	Country	Year	Accession number	Host species
1	HuB/XN	China HuBei	2009	JQ904786	Goat
2	AnH/FD	China AnHui	2011	JQ904787	Goat
3	YN/JS	China YunNan	2011	JQ904788	Goat
4	China vaccine	China GanSu	–	JQ904789	Sheep
5	HuB/XN 2	China HuBei	2010	JQ904790	Goat
6	JS/FX	China JiangSu	2010	JQ904791	Goat
7	SC/JY	China SiChuan	2010	JQ904792	Goat
8	GX/YB	China GuangXi	2011	JQ904793	Goat
9	SD/DY	China ShanDong	2010	JQ904794	Sheep
10	JL/TL	China JiLin	2011	JQ904795	Sheep
11	SC/NC	China SiChuan	2010	JQ904796	Goat
12	CQ/WZ	China ChongQing	2011	JQ904797	Goat
13	SC/YT	China SiChuan	2010	JQ904798	Goat
14	NX/YC	China NingXia	2010	JQ904799	Sheep
15	Hoping	China TW	2008	EU935106	Goat
16	JS04	China	2006	GU903501	Sheep
17	Nantou	China TW	–	DQ904351	Goat
18	Taiping	China TW	–	EU327506	–
19	ORFV/GanSu	China	2009	HQ694772	Sheep
20	Shanxi	China	2009	HQ202153	Goat
21	ORFV/LiaoNing	China	2010	HQ694773	Goat
22	ORFV/HuB	China	2009	GU320351	Goat
23	Jilin	China	2008	FJ808074	Sheep
24	ORFV/Mukteswar/09	India Mukteswar	2009	GU139356	Sheep
25	Muk/2000	India Mukteswar	2000	HM466933	Goat
26	India 67/04	India Izatnagar	2004	DQ263305	Sheep
27	India 79/04	India Mukteswar	2004	DQ263306	Sheep
28	ORFV/2009/Korea	South Korea	2009	GQ328006	Goat
29	Vaccine strain	USA	2003	AY278209	Goat
30	ORFV/USA/ Takin	USA	–	AY424971	Takin
31	ORFV/USA/ Goat	USA	–	AY278208	Goat
32	ORFV/USA/ Sheep	USA	–	AY424970	Sheep
33	D1701	Germany	–	HM133903	Sheep
34	NZ2	New Zealand	2005	DQ184476	–

Numbers 1–14 indicate the strains studied in this paper, whereas the others were downloaded from GenBank

–, unknown

Between 2009 and 2011, clinical samples were collected from 13 cities in 10 Chinese provinces (Fig. 1). Detailed information about the samples is provided in Table 1. In a case from GuangXi (GX/YB), we observed and recorded the clinical symptoms of hoof-type orf, vulva-type orf, and lip-type orf. Scrapings collected from infected goats were suspended in 0.1 M phosphate-buffered saline (1:10 V/V), freeze-thawed twice between −20 °C and 37 °C, and stored overnight at 4 °C. After centrifugation at 5000 rpm for 20 min at 4 °C, DNA was isolated from the supernatant using a genomic DNA purification kit (Promega, USA) and was used as the template in the PCR procedures [13]. Based on the published *B2L* gene sequence, a pair of primers was designed and synthesized (Sangon, China). The PCR products of *B2L* were visualized under ultraviolet light after 1 % agarose gel electrophoresis and ethidium bromide staining. Tissue scrapings from healthy goats were treated the same way and used as negative controls.

**Fig. 1:**
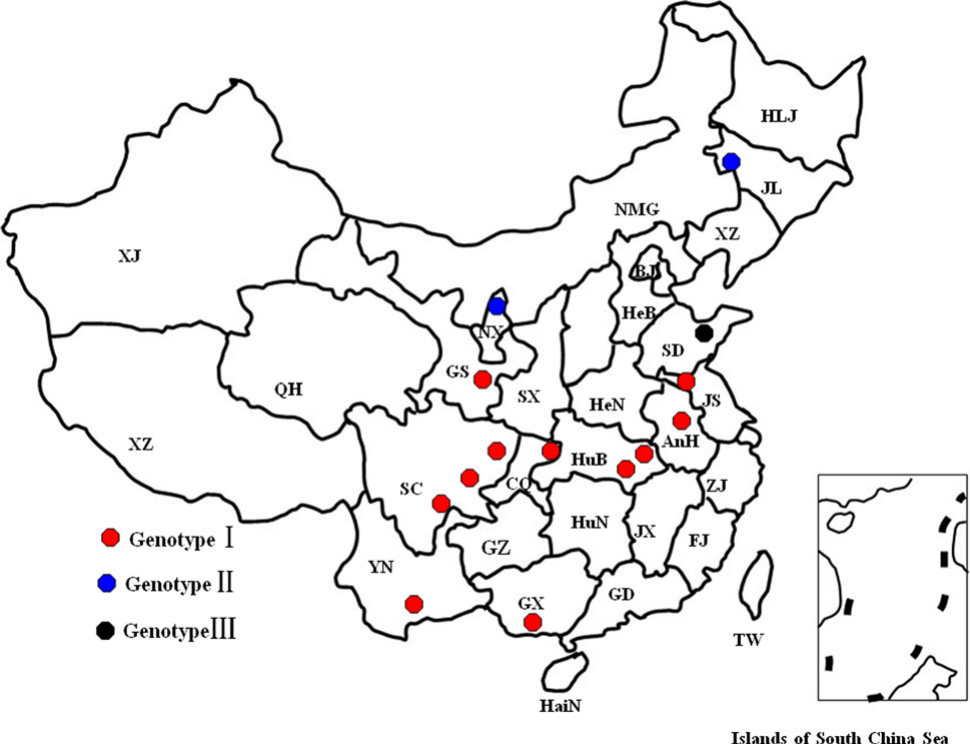
Geographic distribution of the orf cases identified in this study. The dots show the regions in which the orf cases were identified. Red dots, genotype I; blue dots, genotype II; black dots, genotype III (color figure online)

All PCR products were purified using a DNA purification system (Promega) according to the manufacturer’s protocol. The purified PCR products were sequenced using an automated DNA sequencer (Model 3770, Applied Biosystems, USA). The *B2L* gene sequences of ORFV strains from the other countries were obtained from GenBank (http://www.ncbi.nlm.nih.gov/). Sequence editing was performed using the DNASTAR program (http://www.dnastar.com/) [5, 9]. Multiple alignments were produced using the ClustalW program (http://www.clustal.org/) [28]. A phylogenetic tree was constructed based on the deduced amino acid sequences of the *B2L* gene using the neighbor-joining method [25, 35]. Bootstrap analysis was performed for 1000 trials, using the maximum-likelihood method in MEGA version 4.0 (http://www.megasoftware.net/) [27].

Thompson et al. [28] used ClustalW software to uncover possible substitutions in amino acid alignments of the *B2* envelope proteins in wild-type ORFV and attenuated vaccine strains. The Chinese vaccine (JQ904789) and USA vaccine (AY278209) strains were selected for comparison with isolates from goats (JQ904791, JQ904793, AY278208), sheep (JQ904795, JQ904799, AY424970).

Typical clinical symptoms of orf in goats and sheep that were sampled included papules, pimples, ulceration, and incrustation around the lip, hoof, and vulva (see Supplementary material Figure S1). The expected 1,137-bp PCR products were obtained from DNA extracted from scrapings, but not from the negative controls. The sequencing results showed that the *B2L* gene was 1,137 bp long, encoded 378 encoded amino acids, had an average G:C ratio of approximately 63.3 %, and had a predicated molecular weight of 41.7 kDa. The *B2L* gene sequences identified in this study were submitted to NCBI GenBank and assigned accession numbers (JQ904786–JQ904799).

The 14 ORFV isolates from this study and 20 strains downloaded from GenBank were aligned and subjected to phylogenetic analysis. They shared 96.8–98.9 % and 97.5–99.2 % sequence identity at the nucleotide and amino acid level, respectively. The results of neighbor-joining analysis revealed three distinct genotypes (Fig. 2). Genotype I included 17 ORFV strains, 16 of which were from different parts of China and only one of which was from Germany. Genotype II contained nine strains from India (4/9) and China (5/9). Genotype III included eight ORFV isolates from the USA (4/8), New Zealand (1/8), China (2/8), and South Korea (1/8).

**Fig. 2:**
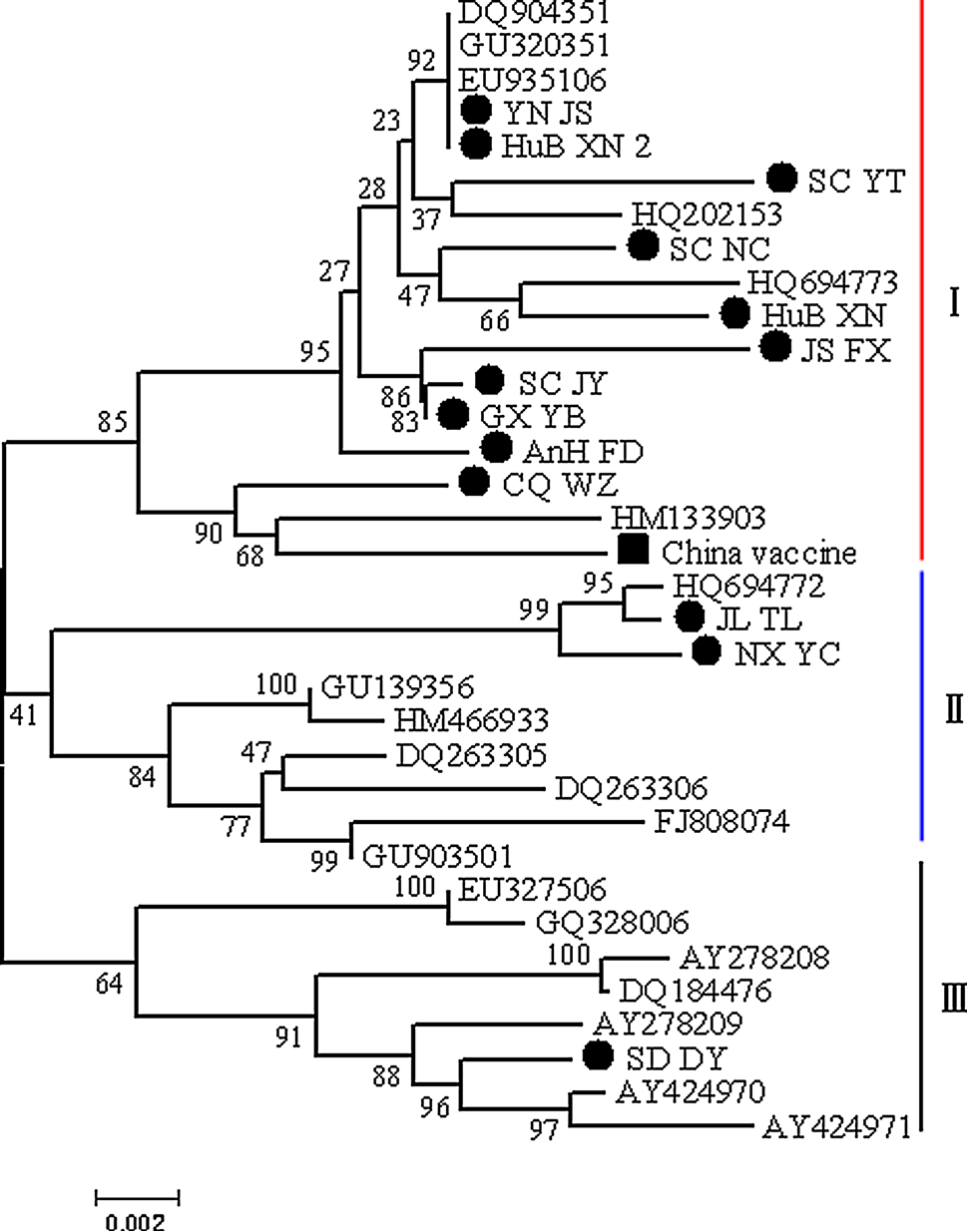
Phylogenetic analysis based on the complete *B2L* gene sequence. The phylogenetic tree was constructed using the neighbor-joining algorithm in MEGA 4.0. I, genotype I (red line); II, genotype II (blue line); III, genotype III (black line). Circular dots represent the wild ORFV strain studied in this paper, while the quadrate dots indicate the vaccine strains identified in this research. The main phylogenetic groups are represented by I (genotype I) and III (genotype III). The black dots indicate the Chinese ORFV strains identified in the current study (color figure online)

In genotype I, 94.1 % (16/17) of the strains were from China; the other (HM133903) was from Germany. Fourteen ORFV strains in genotype I were isolated from Chinese goats. In genotype II, 8/9 (88.9 %) strains (the other was HM466933) were isolated from sheep in India and China. In genotype III, there were two goat strains, two sheep strains, one takin strain, and three host unknown strains.

Multiple alignment of amino acid sequences showed substitutions dispersed all along the length of the protein. Compared with wild ORFV strains, the Chinese vaccine strain JQ904789 had five amino acid substitutions, including A11G, E98A, V101I, S249G, and Q256R (see Supplementary material Figure S2). The USA vaccine strain AY278209 appeared to be divergent from the other strains, as evidenced by substitutions such as S5Y, S6F, V9L, D79N, R111K, and N196D (see Supplementary material Figure S2). For the JS FX goat strain JQ904791, seven unique amino acid substitutions were observed: V16G, A24G, L26R, A27G, N30T, S32T, and T33P. The unique amino acid substitutions V9L and R111K were also found.

It is difficult to differentiate among orf, pox, foot-and-mouth disease, ulcerative dermatosis, dermatophilosis, and staphylococcal dermatitis based on clinical symptoms [31, 32]. The PCR method was able to diagnose ORFV infection in field specimens of the affected animals [16]. It was traditionally believed that clinical symptoms of orf are found around the ovine lips only. Three types (lip, vulva, and hoof) of orf were observed in goats of the GX/YB strain (JQ904793) in this study.

Orf is currently endemic in China. No commercial orf vaccine is available, so the number of outbreaks in sheep and goats continues to increase. Although there have been phylogenetic analyses conducted of orf cases in China [7, 34, 36], there have been limited numbers of case reports in each region. Understanding the molecular epidemiology of an infectious disease is useful for controlling and even eradicating it [4] . In this paper, we identified 14 ORFV strains in China that were distributed among 10 provinces between 2009 and 2011. We first sequenced and compared the *B2L* gene sequence from the attenuated Chinese and USA vaccine strains. The phylogenetic analysis was based on 34 complete *B2L* gene sequences (14 from this study) that had been reported worldwide between 2003 and 2011.

Phylogenetic analysis with 1,000 bootstrap replicates identified three genotypes (Fig. 2). Among the 14 Chinese isolates studied in this paper, 11 belonged to genotype I, two were genotype II, and only the SD/DY (JQ904794) isolate belonged to genotype III. The ORFV strains isolated from one country or nearby regions belonged to similar genotypes, while virus strains from the same species belonged to similar branches (Fig. 2). The middle branch of the phylogenetic tree had a bootstrap value of 41 %. This is a low percentage, and maybe this branch represents a new orf virus genotype (genotype II). The phylogenetic analysis results may indicate the hypothetical source of these viral strains [2, 29], but it is difficult to determine the precise route by which the identified ORFV variants were introduced. This may mean that ORFV strains in China are phylogenetically closely related to the other ORFV strains reported worldwide.

A live attenuated vaccine for orf based on heterologous cells or tissues is effective and popular [14, 21], but its exact attenuated molecular mechanism is obscure. The ORFV glycoprotein is one of the the important target proteins for studying virus-host interactions.

The *B2L* gene has been reported to encode a highly immunogenic envelope protein and play an important role in ORFV immunity [26]. To uncover the differences between vaccine and wild ORFV strains at the amino acid level, eight ORFV strains were selected, and their *B2L* genes were compared using ClustalW software. In the current study, some amino acid substitutions were dispersed along the *B2L* polypeptide of the wild and attenuated vaccine ORFV strains at positions 11, 16, 24, 26, 30, 32, 33, 98, 101, 109, and 313 (see Supplementary material Figure S2). Similar results were reported in India [13] and Brazil [1]. However, no unique amino acid substitutions were observed, which may reflect the fact that ORFV strains are antigenically closely related [11]. The role that these alternative amino acids play in the vaccine strain attenuated process remains to be elucidated. Future studies should produce more detailed epidemiological data about the distribution of ORFV in China and other countries.

## Electronic supplementary material

Below is the link to the electronic supplementary material.

Supplementary material 1 (PDF 54 kb) Figure S1. Representative clinical symptoms of orf virus (ORFV) infection. (A) Goat with severe proliferative ecthyma lesions around the hoof. (B) Severe proliferative ecthyma lesions around the testis and urethral orifice. (C) Wart-like multiple nodules on the upper and lower labia. The arrows indicate the lesion positions

Supplementary material 2 (PDF 29 kb) Figure S2. Multiple sequence alignment of the *B2L* amino acid sequences derived from clinical samples and attenuated vaccine orf virus (ORFV) strains using ClustalW. The dots represent identity among all sequences. The numbers indicate the amino acid positions of the *B2* envelope protein
