# Supplementary figures and images for: Comparison and phylogenetic analysis based on the B2L gene of orf virus from goats and sheep in China during 2009-2011
Source: Arch Virol. 2013 Dec 17;159(6):1475–9. doi: 10.1007/s00705-013-1946-6 (PMC4042016; doi:10.1007/s00705-013-1946-6)

**Figure S1**

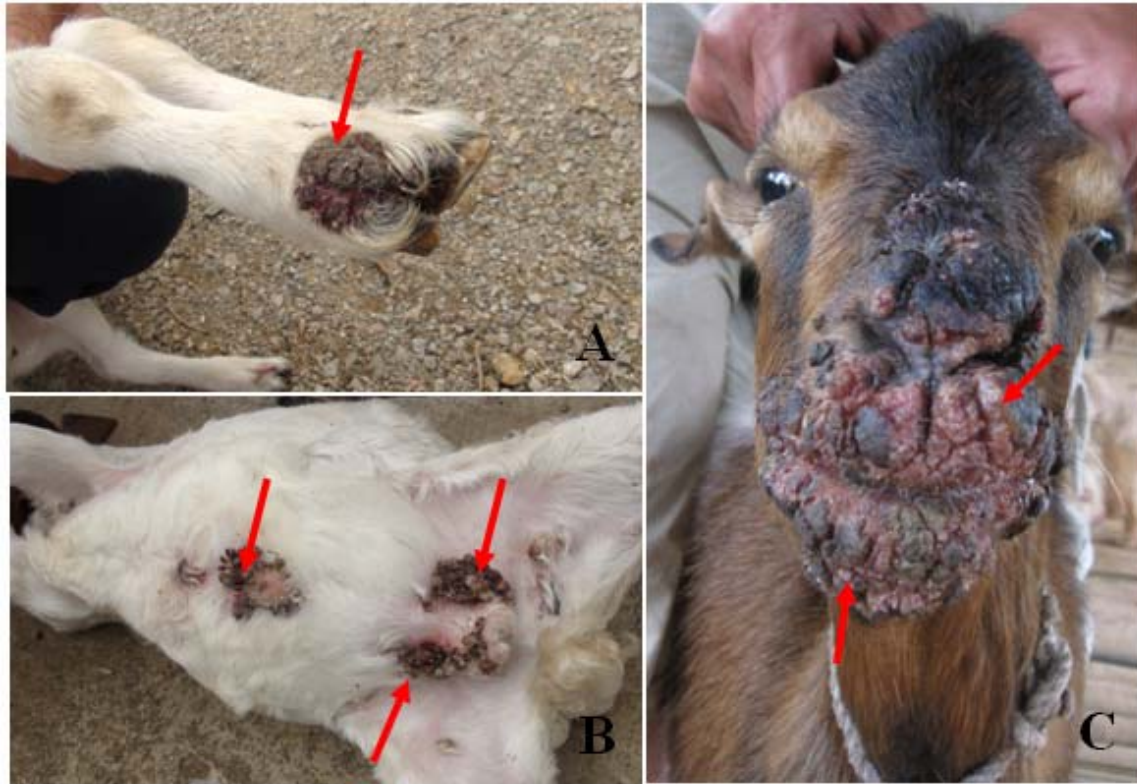

Supplement: Supplementary file 1 — Supplementary material 1 (PDF 54 kb) Figure S1. Representative clinical symptoms of orf virus (ORFV) infection. (A) Goat with severe proliferative ecthyma lesions around the hoof. (B) Severe proliferative ecthyma lesions around the testis and urethral orifice. (C) Wart-like multiple nodules on the upper and lower labia. The arrows indicate the lesion positions [file 705_2013_1946_MOESM1_ESM.pdf]
