# Supplementary material for: Comparison and phylogenetic analysis based on the B2L gene of orf virus from goats and sheep in China during 2009-2011
Source: Arch Virol. 2013 Dec 17;159(6):1475–9. doi: 10.1007/s00705-013-1946-6 (PMC4042016; doi:10.1007/s00705-013-1946-6)
Supplement: Supplementary file 2 — Supplementary material 2 (PDF 29 kb) Figure S2. Multiple sequence alignment of the B2L amino acid sequences derived from clinical samples and attenuated vaccine orf virus (ORFV) strains using ClustalW. The dots represent identity among all sequences. The numbers indicate the amino acid positions of the B2 envelope protein [file 705_2013_1946_MOESM2_ESM.pdf]

Figure S2.

```
               Majority MWPFSSIPVGADCRVVETLPAEVASLAQGNMSTLDCFTAIAESAKKFLYICSFCCNLSSTKEGVDVKDKLCTLAKEGVDV
China vaccine JQ904789 ..........G....................................................................I 80
GX YB goat JQ904793    ................................................................................ 80
JS FX goat JQ904791    ...............G.......G.RG..T.TP............................................... 80
NX YC sheep JQ904799   ...........................H.................................................... 80
JL TL sheep JQ904795   ................................................................................ 80
USA goat AY278208      ........L....................................................................... 80
USA sheep AY424970     ....F...L.....................................................................N. 80
USA vaccien AY278209   ....YF..L.....................................................................N. 80

               Majority TLLVDVQSKDKDADELREAGVNYYKVKVSTREGVGNLLGSFWLSDAGHWYVGSASLTGGSVSTIKNLGLYSTNKHLAWDL
China vaccine JQ904789 .................A..I...........................................................160
GX YB goat JQ904793    ..........................................I..T..................................160
JS FX goat JQ904791    ..........................................I..T..................................160
NX YC sheep JQ904799   ..........................................I.....................................160
JL TL sheep JQ904795   ............................F.............I.....................................160
USA goat AY278208      ..............................K.................................................160
USA sheep AY424970     ..............................K.................................................160
USA vaccien AY278209   ..............................K.................................................160

               Majority MNRYNTFYSMIVEPKVPFTRLCCAVVTPTATNFHLNHSGGGVFFSDSPERFLGFYRTLDEDLVLHRIENAKNSIDLSLLS
China vaccine JQ904789 ................................................................................240
GX YB goat JQ904793    ................................................................................240
JS FX goat JQ904791    ................................................................................240
NX YC sheep JQ904799   ........................I.......................................................240
JL TL sheep JQ904795   ........................I.......................................................240
USA goat AY278208      ........................I..........D............................................240
USA sheep AY424970     ...................................D............................................240
USA vaccien AY278209   ...................................D............................................240

               Majority MVPVIKHASAVEYWPQIIDALLRAAIDRGVRVRVIITEWKNADPLSVSAARSLDDFGVGSVDMSVRKFVVPGRDDAANNT
China vaccine JQ904789 ........G......R................................................................320
GX YB goat JQ904793    ........G......R................................................................320
JS FX goat JQ904791    ........G......R................................................................320
NX YC sheep JQ904799   ..........................N.....................................................320
JL TL sheep JQ904795   ..........................N.....................................................320
USA goat AY278208      ..........................N.............................................G.......320
USA sheep AY424970     ...............R................................................................320
USA vaccien AY278209   ................................................................................320

               Majority KLLIVDDTFAHLTVANLDGTHYRYHAFVSVNAEKGDIVKDLSAVFERDWRSEFCKPIN
China vaccine JQ904789 ..........................................................378
GX YB goat JQ904793    ..........................................................378
JS FX goat JQ904791    ..........................................................378
NX YC sheep JQ904799   ................................Q.........................378
JL TL sheep JQ904795   ..........................................................378
USA goat AY278208      ..........................................................378
USA sheep AY424970     ..........................................................378
USA vaccien AY278209   ..........................................................378
```
